# Joint effects of depressive status and body mass index on the risk of incident hypertension in aging population: evidence from a nationwide population-based cohort study

**DOI:** 10.1186/s12888-023-05105-z

**Published:** 2023-08-19

**Authors:** Qiuxia Luo, Kai Bao, Wenlong Gao, Yuanyuan Xiang, Ming Li, Yuqi Zhang

**Affiliations:** Department of Epidemiology and Health Statistics, School of Public Health, Lanzhou University, No. 222 South Tianshui Road, Lanzhou, Gansu P. R. China

**Keywords:** Depressive symptoms, Depression, BMI: body mass index, Joint effect, Incident hypertension

## Abstract

**Background:**

The impact of depressive status (DS) on hypertension incidence is still controversial and has not been studied in Chinese middle-aged and elderly population. This study aimed to explore the relationship between DS and incident hypertension and analyze the joint effects of DS and body mass index (BMI) on hypertension incidence.

**Methods:**

We conducted a prospective cohort study using data from the China Health and Retirement Longitudinal Study (CHARLS), a nationwide population-based study. In 2013, DS was identified using scores from the 10-item Centre for Epidemiological Studies Depression Scale (CES-D-10) among eligible respondents from CHARLS, and hypertension occurrence was observed until 2018. The multiple Cox models were employed to calculate the associations between DS and hypertension incidence. In addition, we also computed the multiplicative interaction (MI) between DS and BMI of incident hypertension and assessed their additive interaction (AI) through relative excess risk due to interaction (RERI), attributable proportion (AP) or synthetic index (S). Positive AI was indicated by RERI > 0, AP > 0 or S > 1.

**Results:**

Over the 5-year follow-up, depressive symptoms increased the risk of hypertension incidence by 19% (hazard ratio (HR) = 1.19, 95% confidence interval (CI): (1.01, 1.41)), while depression was associated with a 24% increased risk (HR = 1.24; 95% CI: (1.03, 1.50)). Significant MIs between DS and overweight or obesity were observed and almost all of AI indexes showed positive joint effects on incident hypertension, of which the depression-obesity combination had the largest joint effect (RERI = 4.47, 95%CI: (0.28, 8.66); AP = 0.67, 95%CI: (0.50, 0.85); S = 4.86,95%CI: (2.66, 8.86)).

**Conclusion:**

DS could lead to hypertension and this impact was amplified when coexisting with higher BMI. It highlighted a need for precise interventions targeting weight management and depression treatment in the aging population to prevent hypertension.

## Background

Hypertension has become the largest risk factor contributing to the burden of diseases and death [1]. It damages the nervous and cardiovascular systems and can lead to complications such as atherosclerosis, stroke, heart failure and cognitive impairment (https://apps.who.int/iris/bitstream/handle/10665/344424/9789240033986-eng.pdf). Additionally, mental disorders, especially depressive disorder, are a significant cause of morbidity [2], and rank first in the disease burden of mental disorders worldwide [3].

Though extensive researches had been carried out on the relationship between depression and hypertension in different races and populations, there existed the conflicting results. Some studies indicated that the risk of hypertension was multiplied among patients with high scores of depression evaluation, especially major depression [4, 5], while others showed no significant association between depression and the risk of hypertension [6], even decreased incidence of hypertension [7]. Some studies had drawn the positive relationship between depression and hypertension incidence but lacked sufficient statistical power due to a cross-sectional design or relatively small sample size, which required further confirmation [8, 9]. Most cross-sectional studies from prospective researches on depression and hypertension only collected information on exposure and confounders at baseline, which resulted in the loss of important information such as causal order. Furthermore, in studies that had found a link between depression and hypertension, the complex mechanism remained a challenge to understanding, which made it difficult to determine whether depression is causally related to the incident hypertension.

The Chinese population is experiencing rapid aging with a higher prevalence of depression in the middle-aged and elderly population compared to younger groups [10]. Additionally, the prevalence of overweight and obesity are on the rise in the Chinese middle-aged and elderly population [11]. Hypertension involves genetic and behavioral factors, and its etiology is highly age-dependent [12, 13] and weight-dependent [14]. However, there were no studies of the impact of depression on incident hypertension in the middle-aged and elderly Chinese population. Thus, it is needed to highlight and clarify the quantitative relationship between depressive status (DS) and incident hypertension in this group.

Certainly, no studies we have known had focused on the interaction between depression and elevated body mass index (BMI) of incident hypertension, even though the effects of depression and elevated BMI on hypertension had been studied separately. Therefore, we aimed to explore the joint effects of DS and BMI and identify modifiable factors from the perspective of precision medicine to effectively prevent hypertension and its adverse prognosis.

Taking the age-dependent and weight-dependent nature of incident hypertension and depression, and the lack of knowledge about the specific role of BMI level in the association between depression and incident hypertension into consideration, we hypothesized that the association between DS and hypertension onset risk differed in different BMI groups. To test this hypothesis, we analyzed the impact of depression on hypertension incidence in the Chinese middle-aged and elderly population aged 45–80 years using data from the China Health and Retirement Longitudinal Study (CHARLS). Furthermore, we analyzed the joint effects of DS and BMI on incident hypertension to explore possible developmental mechanisms of how DS affects the pathogenesis of hypertension.

## Methods

### Study design and setting

CHARLS is an ongoing nationwide survey, providing an open-access database with representative sample of the middle-aged and elderly population aged ≥ 45 years residing in local communities across China [15]. The survey utilizes face-to-face computer-assisted personal interviews at baseline and is followed by participants every two or three years. The participants come from 450 administrative villages in rural areas and communities in urban areas of 28 provinces in China.

Ethical approval for the study was obtained from the Biomedical Ethical Review Committee of Peking University (IRB00001052-11015; IRB00001052-11014), and written informed consents were obtained from all participants at the beginning of the survey. The survey tools in CHARLS align with international practice, consistent with over 25 Human Resource Specialist (Classification) surveys worldwide.

### Study participants

The study included the individuals aged between 45 and 80 years in 2013. Participants under 45 years due to their lower risk for hypertension and those above 80 years which was well beyond the China’s average life expectancy, and therefore posed a substantial risk of death in the 5-year follow-up period, were excluded. Participants who had hypertension in 2013 were also excluded due to the lack of specific data to determine whether hypertension new onset occurred after the baseline survey date. Ultimately, 11,050 participants were included in the cohort, and all of them were followed up until 2018.

### Depressive status

In the baseline survey, we measured the DS of participants using the 10-item Centre for Epidemiological Studies Depression Scale (CES-D-10), developed by Radloff L. S. from the National Institute of Mental Health [16]. This scale comprises 10 items, with eight items assessing positive symptoms and the remaining two assessing negative symptoms of depression. Participants had 4 responses: rarely or never having the symptoms (< 1 day), some or few times (1–2 days), occasionally or a moderate number of times (3–4 days), and most or all of the time (5–7 days). The total score ranged from 0 to 30, where higher score indicated a higher level of depression. Compared to the original 20-item format, this version demonstrated good test–retest reliability and predictive validity [17]. The CES-D-10 focuses on evaluating the frequency of current depressive symptoms related to emotions or mood, which made it suitable for comparing survey results across different time points. It is widely used in epidemiological investigations to screen participants for further examination and diagnosis. Its reliability and validity in the Chinese population study were confirmed based on the CHARLS data [18]. Participants were categorized into three groups based on their scores from CES-D-10: no depressive symptoms (ND) with scores less than 10, depressive symptoms (DES) with scores between 10 and 14, and depression with scores above 14 [19].

### Identification of hypertension

In 2013, the professionally trained nurses measured the systolic and diastolic blood pressure of the participants using the digital monitor (OMRON HEM-7200) which would be calibrated prior to the measurements. The measurements were taken in a sitting position, within a quiet environment with a stable temperature. Blood pressure was measured three times at least 45 s apart, and the average value of systolic and diastolic blood pressure was recorded for analysis. According to measurements and questionnaires’ self-reports, the criteria for identifying hypertension included: (1) systolic blood pressure ≥ 140 mmHg; (2) diastolic blood pressure ≥ 90 mmHg; (3) self-reported hypertension diagnosed by a doctor; (4) current use of antihypertensive drugs. Blood pressure was not measured in the follow-up survey in 2018 but questionnaires were acquired, so new onset hypertension was identified using the following criteria: (1) self-reported hypertension diagnosed by a doctor between the two surveys; (2) current use of antihypertensive drugs.

**Fig. 1:**
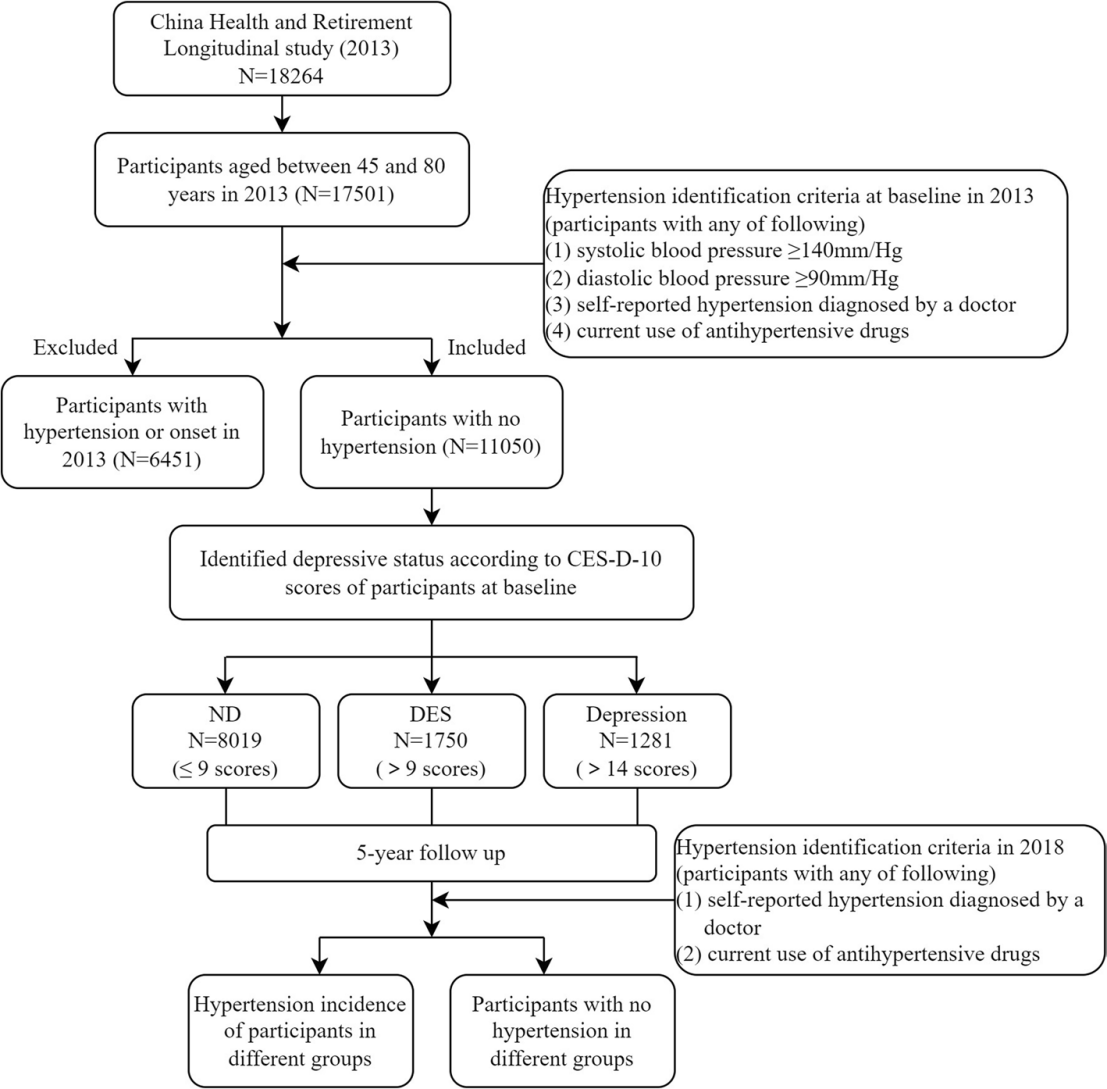
Study flow chat of participants election and follow-up

In the structured questionnaire, some detailed information was collected about the occurrence of hypertension in the 2013 baseline survey and the 2018 follow-up survey, including the situation of self-reported hypertension, the year or age of hypertension occurrence and the use of the hypertensive medicine. The information could be synthetically used to judge the occurrence time of hypertension. Indeed, all the participants at baseline who didn’t meet exclusion criteria were included in the cohort and then followed up in 2018. Figure 1 shows the flow path of selecting and following up all the studied eligible subjects.

### Variables

The information was collected through a structured questionnaire, encompassing sociodemographic factors (age, sex, residence, educational level and marital status), health behaviors (smoking and drinking history), illness history and medication use. Body weight and height were accurately measured to 0.1 kg and 0.1 cm, respectively, and used to calculate BMI (weight in kg divided by squared height in meters). Participants with BMI < 24 kg/m^2^ were categorized as normal or underweight, those with BMI between 24 and 28 kg/m^2^ as overweight and those with BMI ≥ 28 kg/m^2^ as obesity [20]. Age was grouped into three categories: 45 to 50 years, 51 to 60 years and above 60 years. Educational level was classified as elementary school or below, middle school, and high school or above. Sex, residence, marital status, history of smoking, history of drinking, dyslipidemia, diabetes and nephropathy were treated as dichotomous variables. The primary outcome variable of interest in the study was the incidence of hypertension during the follow-up survey.

### Statistical analysis

Categorical variables were described using proportions or rates. The annual incidence rate was computed by the number of new cases within a given year divided by relevant population at risk during the same year from 2014 to2018. Additionally, the 5-year cumulative hypertension incidences in the ND, DES and depression group were calculated using the Kaplan–Meier technique. The study utilized the Cox proportional hazards model to estimate hazard ratios (HRs) and their 95% confidence intervals (CIs) for the association between DS or classified BMI and hypertension incidence. A multiple Cox proportional hazard model was fitted that included sex, age, BMI, history of smoking, history of drinking, dyslipidemia, diabetes, nephropathy, and DS at baseline.

To test the joint effects of DS and BMI on hypertension incidence, interactions with both multiplicative and additive scales were computed. Multiplicative interaction (MI) assesses how the hazard of one exposure factor is multiplied in individuals with the other given risk factors compared to those without some risk factors. The effect of MI can be computed directly by HRs from Cox proportional hazards mode. On the other hand, additive interaction (AI) evaluates the interdependent effect of two factors in producing or preventing an effect and determines whether the joint effect of the two exposures is greater or less than the sum of their respective effects [21, 22].

The relative excess risk due to interaction (RERI), and attributable proportion due to interaction (AP) and synthetic index (S), represent the effect of AI [23]. RERI ≠ 0, AP ≠ 0 or S ≠ 1 indicate a biological AI, which had been applied in previous study [24]. To quantify the amount of interaction with the additive scale, RERI, AP and S were calculated based on the HR estimates. They could be expressed in a Cox interactive model with two dummy variables as follows [23]: RERI = HR_11_ − HR_01_ − HR_10_ + 1, AP = RERI/HR_11_ and S = (HR_11_-1)/[(HR_10_-1) + (HR_01_-1)]. HR_11_ in the formulas denotes the effect of the two dummy variables or the relative risk when a dummy variable coexists with another, HR_01_ denotes the separate effect of the first dummy variable and HR_10_ denotes the separate effect of the second dummy variable. RERI, AP and S were calculated using the regression coefficients and covariance matrix obtained from the Cox model [23]. The delta method by Andersson et al. was used to calculate the 95% CI of RERI, AP and S [23, 25].

Due to the inherent defect of MI to explain the public health implications compared with AI, and the fact that there was sometimes an inconsistency in statistical inference between MI and AI when MI indicates no statistical interactive association [26], in the situation that AI had reflected a biologic interaction better [27], the two types of interaction were considered in the study.

The statistical significance was set to the level of 0.05 in the study. All of the above analyses were carried out by R programming language 4.2.0.(R Core Team (2022). R: A language and environment for statistical computing. R Foundation for Statistical Computing, Vienna, Austria. URL https://www.R-project.org/).

## Results

### Baseline characteristics of respondents

A total of 11,050 participants aged 45–80 years were surveyed in the 2013 baseline survey and followed up for 5 years. Table 1 presents the characteristics of the cohort study participants at baseline. Among all the participants, 65.4% were middle-aged, 48.5% were men, and the majority lived in urban settings and were married. Approximately 80% had received little formal education, while only around 1% were smokers and approximately 3% were drinkers. The prevalence of dyslipidemia and nephropathy slightly exceeded 5% while the prevalence of diabetes was only 3%. Around one-third of the participants were overweighted with BMI but obesity was below 10%. Approximately 16% presented DES while around 12% had developed depression.

**Table 1:** Characteristics of the cohort study participants at baseline

	All	ND	DES	Depression
	*N* = 11,050(%)	*N* = 8019(%)	*N* = 1750(%)	*N* = 1281(%)
Age(years)				
45–50	3126(28.3)	2299(28.7)	497(28.4)	330(25.8)
51–60	4101(37.1)	2973(37.1)	644(36.8)	484(37.8)
> 60	3823(34.6)	2747(34.2)	609(34.8)	467(36.4)
Male	5362(48.5)	4248(53.0)	701(40.0)	413(32.2)
Residence				
Urban	10,069(91.1)	7320(91.3)	1597(91.3)	1152(89.9)
Rural	981(8.9)	699(8.7)	153(8.7)	129(10.1)
Marital status				
Married	9993(90.4)	6775(84.5)	1457(83.3)	986(77.0)
Others	1057(9.6)	1244(15.5)	293(16.7)	295(23.0)
Educational level				
Primary school or below	9409(85.1)	6730(83.9)	1522(87.0)	1157(90.3)
Middle school	916(8.3)	691(8.6)	138(7.9)	87(6.8)
High school or above	725(6.5)	598(7.5)	90(5.1)	37(2.9)
History of smoking	1146(10.4)	868(10.8)	175(10.0)	103(8.0)
History of drinking	3936(35.6)	3018(37.6)	572(32.7)	346(27.0)
Dyslipidaemia	591(5.3)	413(5.2)	101(5.8)	77(6.0)
Diabetes	359(3.2)	241(3.0)	64(3.7)	54(4.2)
Nephropathy	605(5.5)	362(4.5)	121(6.9)	122(9.5)
BMI (kg/m^2^)				
Normal weight/underweight	6425(58.1)	4710(58.8)	1007(57.5)	708(55.3)
Overweight	3957(35.8)	2838(35.4)	629(36.0)	490(38.2)
Obesity	668(6.0)	471(5.8)	114(6.5)	83(6.5)

*BMI* Body mass index, *ND* No depressive symptoms, *DES* Depressive symptoms

### The incidence of hypertension during the follow-up

**Fig. 2:**
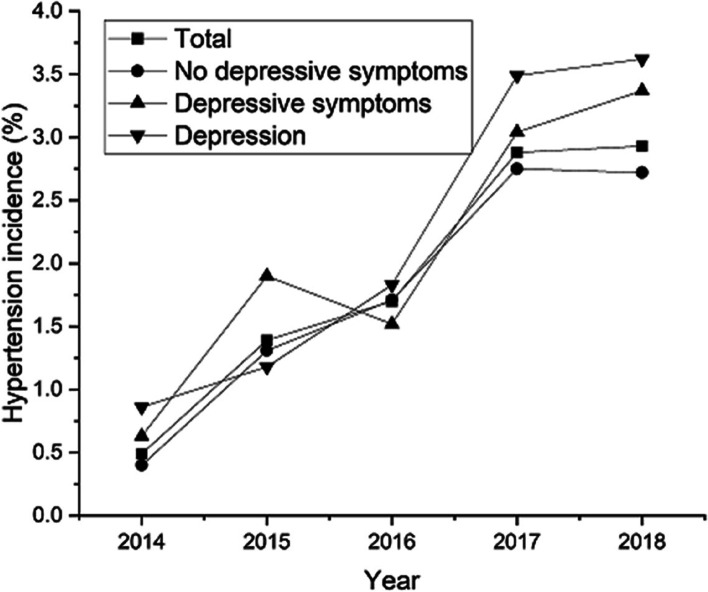
Annual incidence of hypertension in different DS groups from 2014 to 2018

Out of the 11,050 participants, 1001 were identified with new onset of hypertension over a follow-up of five years, resulting in a 5-year incidence of hypertension of 9.06% in the cohort. The incidences of hypertension in participants of ND, exposed to DES and exposed to depression were 8.6% (95% CI: 8.0%, 9.2%), 10.1% (95% CI: 8.6%, 11.5%), and 10.5% (95% CI: 8.9%, 12.2%), respectively. The incidence in the ND group was significantly lower than that in the DES or depression group. Figure 2 illustrates the annual incidence of hypertension in different DS from 2014 to 2018. During the 5-year follow-up, the annual incidence of hypertension in each group showed a tendency to be on the increase annually and the depression group had a higher incidence than DES followed by ND.

### Predictors of hypertension incidence

**Fig. 3:**
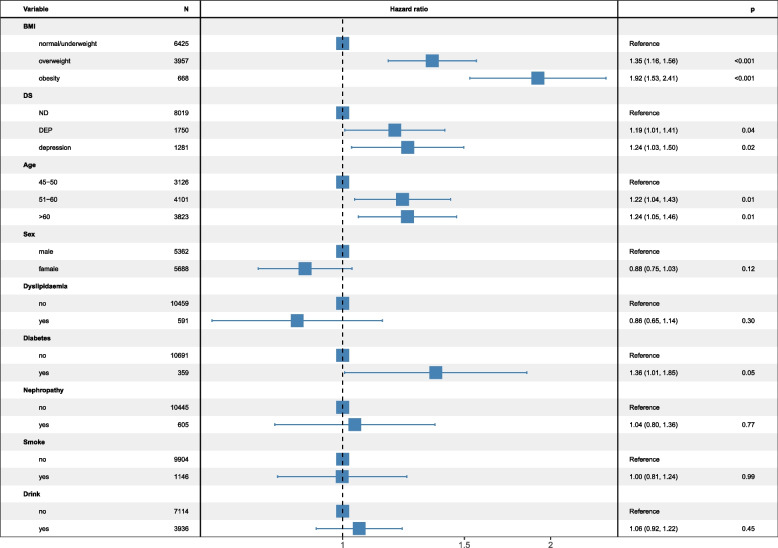
Association between DS and incidence of hypertension using Cox model. DS: depressive status; ND: no depressive symptoms; DES: depressive symptoms; BMI: body mass index

Figure 3 shows the effects of DES or depression and other factors on hypertension incidence in the multiple Cox proportional hazards models. The multivariate analysis revealed that compared with individuals of ND, DES increased the risk of hypertension incidence by 19% (HR = 1.19, 95% CI: (1.01, 1.41)) and depression by 24% (HR = 1.24, 95% CI: (1.03, 1.50)). Moreover, BMI increased this risk with a strong effect than any one of DS.

### The interactions between depressive status and BMI of hypertension incidence

Table 2 depicts the interactions of DS coexisting with BMI on hypertension incidence in the Cox interactive models. The MIs for each combination was statistically significant. Additionally, in a series of AI indexes, almost all indicated the AIs existed, except for RERI of DES and obesity, nevertheless whose AP and S were significant statistically. Particularly, a significant positive AI was detected between depression and obesity of hypertension incidence (RERI = 4.47, 95% CI: (0.28, 8.66); AP = 0.67, 95% CI: (0.50, 0.85); S = 4.86, 95% CI: (2.66, 8.86)). In other words, the hypertension incidence in individuals with obesity and depression was 4.47 times higher than that of normal/underweight individuals of ND. Additionally, approximately 67% of the risk of hypertension was attributable to the interaction between these two exposures.

**Table 2:** Associations and interactions between depressive status and BMI of hypertension incidence^a^

	Multiplicative interaction	Additive interaction		
	HR (95%CI)	RERI (95%CI)	AP (95%CI)	S(95%CI)
**Model DES**				
BMI				
overweight	1.68(1.29,2.19)	1.13(0.17,2.09)	0.43(0.26,0.59)	3..18(2.10,4.82)
obesity	1.94(1.15,3.26)	2.28(-0.37,4.94)	0.52(0.26,0.78)	3.08(1.56,6.07)
**Model Depression**				
BMI				
overweight	1.60(1.19,2.17)	1.08(0.03,2.14)	0.41(0.21,0.60)	2.88(1.81,4.57)
obesity	2.79(1.68,4.63)	4.47(0.28,8.66)	0.67(0.50,0.85)	4.86(2.66,8.86)

*BMI* Body mass index, *DES* Depressive symptoms, *RERI* Relative excess risk due to interaction, *AP* Attributable proportion, *S* Synergy index

^a^Models were adjusted for age, sex, dyslipidaemia, diabetes, nephropathy, history of smoking, history of drinking

## Discussion

To our knowledge, the current study was the first large longitudinal study to use nationally representative data to assess not only the association between DS and hypertension incidence but also the joint effects of DS and BMI in a middle-aged and elderly Chinese population. Our findings suggested that exposure to DES or depression was associated with a higher risk of incident hypertension. Specifically, DES increased the risk by 19% (HR = 1.19, 95%CI: (1.01,1.41)) and depression by 24% (HR = 1.24, 95%CI: (1.03–1.50)), even after possible confounders were adjusted. The risk of hypertension incidence demonstrated a growing trend with the depressive severity. Moreover, being overweight or obese in the elderly significantly enhanced this association. Considering the joint effect of depression and obesity on hypertension incidence, it is crucial to aggressively control weight within a reasonable range and provide health education for the elderly population. Furthermore, an increase in mental health services should be prioritized through the general and comprehensive care facilities, specialized mental health care, human resources, and complementary and alternative medicine departments.

In contrast, our results differed slightly from those reported by Delaney JA et al. which indicated DES lacked inferential ability about incident hypertension [28]. However, our study, with a large cohort of 11,050 participants from CHARLS, representing a large population, provided robust evidence for inferring the causal relationship between DS and hypertension incidence. Moreover, underlying pathologic and physiologic mechanisms strongly supported this relationship. Depressive episodes indirectly stimulated the release of adrenocorticotropic hormone and cortisol, increasing the risk of hypertension [29, 30]. Additionally, depression may lead to hyperfunctioning of the sympathetic and parasympathetic nervous systems, resulting in elevated blood pressure due to increased catecholamine secretion and myocardial contractility [31]. Furthermore, depression may activate the renin-angiotensin system, further elevating blood pressure [32, 33]. Sleep disorders caused by depression also contributed to cardiovascular dysfunction [34]. Some animal experiments had shown that sleep deprivation and intermittent hypoxia in the rodent sleep APNEA models could induce an acute increase in reactive oxygen species and decrease nitric oxide release, while the transcription factors that were the basis of the antioxidant defense mechanism had also changed [35, 36]. In addition, substantial changes in behavior and lifestyle in depressed patients made the prevention of hypertension very challenging [37–39]. Our study highlighted the direct positive effects of DES or depression on incident hypertension, providing valuable insights for addressing this public health issue. Early treatment of depression is essential to enable patients to better assess and manage health problems, potentially mitigating the risk of hypertension [40].

Our study revealed that both DES and depression combined with higher BMI increased the risk of hypertension. Epidemiologic studies had consistently shown a strong bidirectional relationship between depression and obesity, which could explain the joint role of these factors in hypertension [41, 42].

Several hypotheses could be offered to explain the joint effect of depression and high BMI on incident hypertension in the study. First, peripheral inflammation plays a crucial role in this joint effect, as both depression and obesity are associated with a pro-inflammatory status and higher oxidative stress. Adipose tissue, known to be able to mediate these biological changes, regulates inflammatory responses systemically and locally [43–45]. Obesity leads to changes in adipocyte secretion, resulting in the production of pro-inflammatory cytokines. Meanwhile, episodes of depression are often accompanied by biological abnormalities related to overweight and obesity, such as insulin resistance, mitochondrial dysfunction, and increased oxidative status [46]. Additionally, the joint effects of depression and obesity can enhance the aging process through the modulation of aging-related secretory phenotypes [47].

Second, neuroendocrinological evidence supported another hypothesis. Obesity leads to the accumulation of senescent glial cells near the lateral ventricle, contributing to depression-related behavior [44]. Neural circuits related to obesity and mental disorders, such as melanocortin 4 receptor neurons, play a role in regulating mentally-associated weight gain by receiving GABAergic (GABA: gamma-aminobutyric acid) projections from hypothalamic agouti-related peptide (AgRP) neurons onto α 5-containing GABAA (gamma-aminobutyric acid type A) receptors and serotonergic afferents onto 5-Hydroxytryptamine type 3 receptors. Mice fed with a high-fat diet become not only obese but also anxious or depressed due to defects in brain circuits. Correcting these specific interferences genetically or pharmacologically can reduce depression and weight, thereby affecting eating preferences [48]. Moreover, the leptin-melanocortin pathway, related to obesity [49], has recently been proposed to be involved in depression [50].

Third, genetic defects also contributed to the joint effect. Deletion of the brain-derived neurotrophic factor (BDNF) gene in specific brain regions leads to impaired GABAergic transmission and increased anxiety [51]. On the other hand, deletion or interruption of BDNF-TrkB signaling results in significant hyperphagocytosis and severe obesity in mice [52]. Additionally, perturbation of fat mass and obesity-associated (FTO) enzyme activity disrupts genes involved in energy metabolism, leading to dysregulation of energy and adipose tissue homeostasis in mice [53]. The FTO research had been the recent discovery of the first N6-methyladenosine (m6A) ribonucleic acid demethylase, which has been linked to depression, and its gene polymorphisms show a positive correlation with depression subtypes, such as atypical depression characterized by increased appetite, sleepiness and weight gain by regulating RNA (Ribonucleic acid) processing [54, 55]. These proved that DS and higher BMI could independently contribute to the risk of hypertension, while they also promoted each other’s progress, jointly increasing the risk of incident hypertension. Therefore, effective management of overweight or obesity and its associated comorbidities is essential for the middle-aged and elderly population, requiring a thorough understanding of the underlying biological mechanisms.

Our study had several limitations. Firstly, in 2018, we did not have access to directly-measured blood pressure data, so we relied on a combination of self-reported professional diagnoses of hypertension and self-reported current use of anti-hypertensive drugs to identify hypertension during the follow-up until 2018. Nevertheless, the validity of self-reported hypertension had been shown to be high in previous studies, with sensitivity and specificity of 83% and 81%, respectively [56]. Secondly, as an observational cohort study, we controlled many observed possible risk factors of hypertension while assessing the causal relationship between DES or depression on hypertension. However, it was important to acknowledge that there might be unmeasured or unknown factors that could still confound our findings. Lastly, compared to some previous cohort studies, the duration of our follow-up was relatively short. Nevertheless, the time-window was still sufficient for us to infer the causal relationship [57, 58].

## Conclusion

In conclusion, the risk of hypertension incidence increased with the severity of depression. Moreover, the joint effect of DS and higher BMI positively contributed to the additional risk of incident hypertension. Therefore, monitoring weight status and providing effective DS treatment are crucial preventive measures to alleviate hypertension in the aging Chinese population.

## Data Availability

The database was used from China Health and Retirement Longitudinal Study. (http://charls.pku.edu.cn/).

## Abbreviations

DS: Depressive status
BMI: Body mass index
CES-D-10: 10-Item Centre for Epidemiological Studies Depression Scale
CHARLS: China Health and Retirement Longitudinal Study
MI: Multiplicative interaction
RERI: Relative excess risk due to interaction
AP: Attributable proportion
S: Synthetic index
AI: Additive interaction
HR: Hazard ratio
CI: Confidence interval
ND: No depressive symptoms
DES: Depressive symptoms
GABA: Gamma-aminobutyric acid
AgRP: Agouti-related peptide
GABAA: Gamma-aminobutyric acid type A
BDNF: Brain-derived neurotrophic factor
TrkB: Tyrosine kinase receptor B
FTO: Fat mass and obesity associated
m6A: N6-methyladenosine
RNA: Ribose nucleic acid

## References

[1] Beaney T, Burrell LM, Castillo RR, Charchar FJ, Cro S, Damasceno A, Kruger R, Nilsson PM, Prabhakaran D, Ramirez AJ (2019). May Measurement Month 2018: a pragmatic global screening campaign to raise awareness of blood pressure by the International Society of Hypertension. Eur Heart J.

[2] Schaakxs R, Comijs HC, Lamers F, Kok RM, Beekman ATF, Penninx B (2018). Associations between age and the course of major depressive disorder: a 2-year longitudinal cohort study. Lancet Psychiatry.

[3] GBD 2019 Diseases and Injuries Collaborators. Global burden of 369 diseases and injuries in 204 countries and territories, 1990-2019: a systematic analysis for the Global Burden of Disease Study 2019. Lancet. 2020;396(10258):1204–22. 10.1016/S0140-6736(20)30925-9. Erratum in: Lancet. 2020;396(10262):1562. 10.1016/S0140-6736(20)30925-9 PMC7567026 33069326

[4] Collazos-Perdomo D, Ramirez-Ramos CF, Torres de Galvis MY, Correas-Orozco L, Ramirez-Mendez D, CastillaAgudelo GA, Martinez Cano CA, Gallego C, Saldarriaga C (2020). [Association between major depression and arterial hypertension in a Colombian population]. Hipertens Riesgo Vasc.

[5] Patten SB, Beck CA, Kassam A, Williams JV, Barbui C, Metz LM (2005). Long-term medical conditions and major depression: strength of association for specific conditions in the general population. Can J Psychiatry.

[6] Wiehe M, Fuchs SC, Moreira LB, Moraes RS, Pereira GM, Gus M, Fuchs FD (2006). Absence of association between depression and hypertension: results of a prospectively designed population-based study. J Hum Hypertens.

[7] Hildrum B, Mykletun A, Holmen J, Dahl AA (2008). Effect of anxiety and depression on blood pressure: 11-year longitudinal population study. Br J Psychiatry.

[8] Cramer H, Lauche R, Adams J, Frawley J, Broom A, Sibbritt D (2020). Is Depression associated with unhealthy behaviors among middle-aged and older women with hypertension or heart disease?. Womens Health Issues.

[9] Flórez-García V, Rojas-Bernal L, Bareño-Silva J (2020). Depression and sleep disorders related to hypertension: a cross-sectional study in Medellín, Colombia. Rev Colomb Psiquiatr (Engl Ed).

[10] Wang Z, Chen Z, Zhang L, Wang X, Hao G, Zhang Z, Shao L, Tian Y, Dong Y, Zheng C (2018). Status of hypertension in China: results from the China hypertension survey, 2012–2015. Circulation.

[11] Pan XF, Wang L, Pan A (2021). Epidemiology and determinants of obesity in China. Lancet Diabetes Endocrinol.

[12] Delgado J, Bowman K, Ble A, Masoli J, Han Y, Henley W, Welsh S, Kuchel GA, Ferrucci L, Melzer D (2018). Blood pressure trajectories in the 20 years before death. JAMA Intern Med.

[13] Worldwide trends in blood pressure from 1975 to 2015: a pooled analysis of 1479 population-based measurement studies with 19·1 million participants. Lancet. 2017;389:37-55. 10.1016/s0140-6736(16)31919-5. 10.1016/S0140-6736(16)31919-5 PMC5220163 27863813

[14] Nyamdorj R, Qiao Q, Lam TH, Tuomilehto J, Ho SY, Pitkäniemi J, Nakagami T, Mohan V, Janus ED, Ferreira SR (2008). BMI compared with central obesity indicators in relation to diabetes and hypertension in Asians. Obesity (Silver Spring).

[15] Zhao Y, Hu Y, Smith JP, Strauss J, Yang G (2014). Cohort profile: the China Health and Retirement Longitudinal Study (CHARLS). Int J Epidemiol.

[16] Andresen EM, Malmgren JA, Carter WB, Patrick DL (1994). Screening for depression in well older adults: evaluation of a short form of the CES-D (Center for Epidemiologic Studies Depression Scale). Am J Prev Med.

[17] Carpenter JS, Andrykowski MA, Wilson J, Hall LA, Rayens MK, Sachs B, Cunningham LL (1998). Psychometrics for two short forms of the Center for Epidemiologic Studies-Depression Scale. Issues Ment Health Nurs.

[18] Lei X, Sun X, Strauss J, Zhang P, Zhao Y (2014). Depressive symptoms and SES among the mid-aged and elderly in China: evidence from the China Health and Retirement Longitudinal Study national baseline. Soc Sci Med.

[19] Qin X, Wang S, Hsieh C-R (2018). The prevalence of depression and depressive symptoms among adults in China: estimation based on a National Household Survey. China Econ Rev.

[20] Zhou BF (2002). Predictive values of body mass index and waist circumference for risk factors of certain related diseases in Chinese adults–study on optimal cut-off points of body mass index and waist circumference in Chinese adults. Biomed Environ Sci.

[21] Kalilani L, Atashili J (2006). Measuring additive interaction using odds ratios. Epidemiol Perspect Innov.

[22] de Mutsert R, Jager KJ, Zoccali C, Dekker FW (2009). The effect of joint exposures: examining the presence of interaction. Kidney Int.

[23] Andersson T, Alfredsson L, Källberg H, Zdravkovic S, Ahlbom A (2005). Calculating measures of biological interaction. Eur J Epidemiol.

[24] Yang X, So WY, Ma RC, Kong AP, Lee HM, Xu G, Ozaki R, Chan JC (2012). Synergistic effects of low LDL cholesterol with other factors for the risk of cancer in type 2 diabetes: the Hong Kong Diabetes Registry. Acta Diabetol.

[25] Hosmer DW, Lemeshow S (1992). Confidence interval estimation of interaction. Epidemiology.

[26] Rod NH, Lange T, Andersen I, Marott JL, Diderichsen F (2012). Additive interaction in survival analysis: use of the additive hazards model. Epidemiology.

[27] Rothman KJGS, Lash TL (2008). Modern epidemiology.

[28] Delaney JA, Oddson BE, Kramer H, Shea S, Psaty BM, McClelland RL (2010). Baseline depressive symptoms are not associated with clinically important levels of incident hypertension during two years of follow-up: the multi-ethnic study of atherosclerosis. Hypertension.

[29] Brown ES, Varghese FP, McEwen BS (2004). Association of depression with medical illness: does cortisol play a role?. Biol Psychiatry.

[30] Torpy DJ, Mullen N, Ilias I, Nieman LK (2002). Association of hypertension and hypokalemia with Cushing's syndrome caused by ectopic ACTH secretion: a series of 58 cases. Ann N Y Acad Sci.

[31] Won E, Kim YK (2016). Stress, the autonomic nervous system, and the immune-kynurenine pathway in the etiology of depression. Curr Neuropharmacol.

[32] Ho AK, Thorpe CT, Pandhi N, Palta M, Smith MA, Johnson HM (2015). Association of anxiety and depression with hypertension control: a US multidisciplinary group practice observational study. J Hypertens.

[33] Thailer SA, Friedman R, Harshfield GA, Pickering TG (1985). Psychologic differences between high-, normal-, and low-renin hypertensives. Psychosom Med.

[34] Göder R, Hinrichsen I, Seeck-Hirschner M, Pfeiffer R, Weinhold SL, Baier PC, Hanss R, Schulz-DuBois C (2016). Sleep at baseline and after electroconvulsive therapy in patients with major depression. Psychiatry Res.

[35] Villafuerte G, Miguel-Puga A, Rodríguez EM, Machado S, Manjarrez E, Arias-Carrión O (2015). Sleep deprivation and oxidative stress in animal models: a systematic review. Oxid Med Cell Longev.

[36] Ibrahim AY, ChamsiBasha A, Saquib J, Zaghloul MS, Al-Mazrou A, Saquib N (2019). Sleep duration is associated with depressive symptoms among expatriate nurses. J Affect Disord.

[37] Galper DI, Trivedi MH, Barlow CE, Dunn AL, Kampert JB (2006). Inverse association between physical inactivity and mental health in men and women. Med Sci Sports Exerc.

[38] John U, Meyer C, Rumpf HJ, Hapke U (2004). Smoking, nicotine dependence and psychiatric comorbidity–a population-based study including smoking cessation after three years. Drug Alcohol Depend.

[39] Patten SB, Charney DA (1998). Alcohol consumption and major depression in the Canadian population. Can J Psychiatry.

[40] Hare DL, Toukhsati SR, Johansson P, Jaarsma T (2014). Depression and cardiovascular disease: a clinical review. Eur Heart J.

[41] Luppino FS, de Wit LM, Bouvy PF, Stijnen T, Cuijpers P, Penninx BW, Zitman FG (2010). Overweight, obesity, and depression: a systematic review and meta-analysis of longitudinal studies. Arch Gen Psychiatry.

[42] Mannan M, Mamun A, Doi S, Clavarino A (2016). Prospective associations between depression and obesity for adolescent males and females- a systematic review and meta-analysis of longitudinal studies. PLoS ONE.

[43] van Agtmaal MJM, Houben A, Pouwer F, Stehouwer CDA, Schram MT (2017). Association of microvascular dysfunction with late-life depression: a systematic review and meta-analysis. JAMA Psychiat.

[44] Ogrodnik M, Zhu Y, Langhi LGP, Tchkonia T, Krüger P, Fielder E, Victorelli S, Ruswhandi RA, Giorgadze N, Pirtskhalava T (2019). Obesity-induced cellular senescence drives anxiety and impairs neurogenesis. Cell Metab.

[45] Kershaw EE, Flier JS (2004). Adipose tissue as an endocrine organ. J Clin Endocrinol Metab.

[46] Diniz BS, Fisher-Hoch S, McCormick J (2018). The association between insulin resistance, metabolic variables, and depressive symptoms in Mexican-American elderly: a population-based study. Int J Geriatr Psychiatry.

[47] Diniz BS, Reynolds Iii CF, Sibille E, Bot M, Penninx B (2019). Major depression and enhanced molecular senescence abnormalities in young and middle-aged adults. Transl Psychiatry.

[48] Xia G, Han Y, Meng F, He Y, Srisai D, Farias M, Dang M, Palmiter RD, Xu Y, Wu Q (2021). Reciprocal control of obesity and anxiety-depressive disorder via a GABA and serotonin neural circuit. Mol Psychiatry.

[49] O'Rahilly S, Farooqi IS (2008). Human obesity: a heritable neurobehavioral disorder that is highly sensitive to environmental conditions. Diabetes.

[50] Guo M, Huang TY, Garza JC, Chua SC, Lu XY (2013). Selective deletion of leptin receptors in adult hippocampus induces depression-related behaviours. Int J Neuropsychopharmacol.

[51] Xie X, Yang H, An JJ, Houtz J, Tan JW, Xu H, Liao GY, Xu ZX, Xu B (2019). Activation of anxiogenic circuits instigates resistance to diet-induced obesity via increased energy expenditure. Cell Metab.

[52] An JJ, Liao GY, Kinney CE, Sahibzada N, Xu B (2015). Discrete BDNF neurons in the paraventricular hypothalamus control feeding and energy expenditure. Cell Metab.

[53] Hess ME, Hess S, Meyer KD, Verhagen LA, Koch L, Brönneke HS, Dietrich MO, Jordan SD, Saletore Y, Elemento O (2013). The fat mass and obesity associated gene (Fto) regulates activity of the dopaminergic midbrain circuitry. Nat Neurosci.

[54] Meyer KD, Saletore Y, Zumbo P, Elemento O, Mason CE, Jaffrey SR (2012). Comprehensive analysis of mRNA methylation reveals enrichment in 3' UTRs and near stop codons. Cell.

[55] Milaneschi Y, Lamers F, Mbarek H, Hottenga JJ, Boomsma DI, Penninx BW (2014). The effect of FTO rs9939609 on major depression differs across MDD subtypes. Mol Psychiatry.

[56] Martin LM, Leff M, Calonge N, Garrett C, Nelson DE (2000). Validation of self-reported chronic conditions and health services in a managed care population. Am J Prev Med.

[57] Jonas BS, Franks P, Ingram DD (1997). Are symptoms of anxiety and depression risk factors for hypertension? Longitudinal evidence from the National Health and Nutrition Examination Survey I Epidemiologic Follow-up Study. Arch Fam Med.

[58] Nabi H, Chastang JF, Lefèvre T, Dugravot A, Melchior M, Marmot MG, Shipley MJ, Kivimäki M, Singh-Manoux A (2011). Trajectories of depressive episodes and hypertension over 24 years: the Whitehall II prospective cohort study. Hypertension.

